# BCR: a promiscuous fusion partner in hematopoietic disorders

**DOI:** 10.18632/oncotarget.26837

**Published:** 2019-04-12

**Authors:** Malalage N. Peiris, Fangda Li, Daniel J. Donoghue

**Affiliations:** ^1^ Department of Chemistry and Biochemistry, University of California San Diego, La Jolla, California, USA; ^2^ UCSD Moores Cancer Center, University of California San Diego, La Jolla, California, USA

**Keywords:** oncogenic fusion protein, chromosomal translocation, leukemia, breakpoint cluster region, RTK

## Abstract

Considerable advances have been made in our understanding of the molecular basis of hematopoietic cancers. The discovery of the BCR-ABL fusion protein over 50 years ago has brought about a new era of therapeutic progress and overall improvement in patient care, mainly due to the development and use of personalized medicine and tyrosine kinase inhibitors (TKIs). However, since the detection of BCR-ABL, BCR has been identified as a commonly occurring fusion partner in hematopoietic disorders. BCR has been discovered fused to additional tyrosine kinases, including: Fibroblast Growth Factor Receptor 1 (FGFR1), Platelet-derived Growth Factor Receptor Alpha (PDGFRA), Ret Proto-Oncogene (RET), and Janus Kinase 2 (JAK2). While BCR translocations are infrequent in hematopoietic malignancies, clinical evidence suggests that patients who harbor these mutations benefit from TKIs and additional personalized therapies. The improvement of further methodologies for characterization of these fusions is crucial to determine a patient’s treatment regimen, and optimal outcome. However, potential relapse and drug resistance among patients’ highlights the need for additional treatment options and further understanding of these oncogenic fusion proteins. This review explores the mechanisms behind cancer progression of these BCR oncogenic fusion proteins, comparing their similarities and differences, examining the significance of BCR as a partner gene, and discussing current treatment options for these translocation-induced hematopoietic malignancies.

## INTRODUCTION: CHROMOSOMAL TRANSLOCATIONS IN CANCER

Cancer arises from genetic alterations consisting of gene mutation, gene over-activation or gene loss of function. In the last 60 years, chromosomal translocations that encode for functional oncogenic proteins have been identified in numerous cancer types, and account for approximately 20% of all malignant neoplasms [1]. With the emergence of personalized medicine and cancer genome sequencing, the characterization of mutations such as chromosomal translocations is vital. Translocations usually arise from multiple DNA double strand breaks (DSB) in chromosomes that can occur for various reasons. Illegitimate V(D)J recombination, class switch recombination, homologous recombination, non-homologous end joining, and genome fragile sites are all suggested to produce chromosomal translocations [2]. However, the presence of a translocation is not always a hallmark of cancer [3]. Previous studies have found leukemogenic translocations in the blood of healthy individuals, indicating that translocations alone may not be sufficient to produce malignant cells. Instead, these translocations produce pre-malignant cells, which may require additional mutations for cancer to occur [2–4].

Identified chromosomal translocations are numerous and varied, many of which produce a translatable fusion protein with oncogenic potential. However, a common theme amongst these fusions is the contribution of a dimerization domain by a partner gene, often fused to a kinase [5]. Arguably the most well studied oncogenic fusion, breakpoint cluster region-Abelson murine leukemia viral oncogene 1 (BCR-ABL), discovered in 1960 and found in 95% of chronic myeloid leukemia cases (CML), is the archetype of this theme. The BCR-ABL translocation is thus referred to as the Philadelphia chromosome, and resulting leukemias are referred to as Ph+ leukemias. Since its original discovery as part of the Philadelphia chromosome, BCR has been identified fused to multiple tyrosine kinases, including FGFR1, PDGFRA, RET, and JAK2 in hematopoietic malignancies [5]. Yet, the underlying reason behind the commonality of BCR as a fusion partner is not well understood. It has been speculated that genes such as BCR are located near chromosomal fragile sites. These sites are specific genomic regions that show gaps or breaks on metaphase chromosomes due to replication stress which are prone to breakage and translocation as a result. Indeed, 64% of breakpoints in chromosomal translocations implicated in hematological malignancies correspond to common fragile sites, and may account for the increased frequency of BCR as a fusion partner in hematopoietic neoplasms [6]. Furthermore, BCR-ABL positive CML is a leukemic stem cell disease, where CML is maintained by a population of leukemic stem cells, that are capable of cell colonization [7, 8]. Although BCR fusions have been detected in solid tumors, BCR fusion proteins that are drivers of cancer have solely been identified in hematological cancers to date [9]. BCR is highly expressed hematopoietic tissue, which may account for its function as a fusion partner in blood cancers (Table 1) [10].

**Table 1:** Commonly occurring BCR fusion proteins in hematopoietic cancers

Translocation	Breakpoints	Cancer type	Frequency	Localization	Treatment	Ref
BCR-ABL	t(9;22) (q34;q11)	CML ALL AML Neutrophilic CML	1.8:100,000	cytoplasmic	Imatinib Ponatinib Dasatinib Nilotinib Bosutinib Aminoxyrone HSCT CAR-T Blinatumomab	[16, 29, 32, 81, 85, 88, 89]
BCR-FGFR1	t(8;22) (p11;q11)	EMS SCLL aCML AML B-cell lymphoma	<100 to date	cytoplasmic	Ponatinib Dovitinib Dasatinib HSCT	[33, 39, 40]
BCR-PDGFRA	t(4;22) (q12;q11)	aCML T-cell Lymphoblastic Leukemia	<100 to date	unknown	Imatinib	[16, 47, 90]
BCR-RET	t(10;22) (q11;q11)	aCML CMML	<100 to date	cytoplasmic	Sorafenib	[50, 91]
BCR-JAK2	t(9;22)(p24;q11)	aCML AML ALL	<100 to date	cytoplasmic	TG101209 Ruxolitinib HSCT	[52, 54, 55, 92–94]

Here we present a timely review, which examines the importance of BCR as a translocation partner in hematopoietic cancers. The commonality of BCR as a fusion partner will be addressed and the molecular mechanisms of these BCR fusions will be discussed in detail, along with current treatment options and patient outcomes for cancers positive for these fusions (Figure 2). Furthermore, BCR has been uncovered as a fusion partner in 19 additional translocations found in various cancers [9] (Table 2). However, the biological activity of the resulting fusion proteins, if any, and the potential importance of BCR in these translocations is unknown. The discovered oncogenic BCR fusions once again highlight the importance of determining malignant genetic alterations in patients, and a need for personalized medical treatments.

**Table 2:** Additional BCR fusions found in cancers

Translocation	Breakpoint	Cancer type	Reference
**ABL1-BCR**	t(9;22)(q34;q11)	CML	[95, 96]
**BCR-CYYR1**	t(21;22)(q21;q11)	Not reported	[88, 96]
**BCR-GNAZ**	(22;22)(q11;q11)	Squamous cell carcinoma	[96, 97]
**BCR-GOLPH3L**	t(1;22)(q21;q11)	Not reported	[88, 96]
**BCR-LOC220729**	t(3;22)(q29;q11)	Not reported	[88, 96]
**BCR-MOV10L1**	(22;22)(q13;q11)	Breast: Adenocarcinoma	[96, 98]
**BCR-MRVI1**	t(11;22)(p15;q11)	Breast: Adenocarcinoma	[96, 98]
**BCR-MTHFS**	t(15;22)(q25;q11)	Not reported	[88, 96]
**BCR-MTTP**	t(4;22)(q23;q11)	Not reported	[88, 96]
**BCR-PI4KA**	(22;22)(q11;q11)	Head and Neck	[96, 99]
**BCR-RALGPS1**	t(9;22)(q33;q11)	ALL	[88, 96]
**BCR-SET**	t(9;22)(q34;q11)	Not reported	[96, 100]
**BCR-TOM1**	(22;22)(q12;q11)	Mouth-Oropharynx: Squamous cell carcinoma	[96, 98]
**BCR-UPB1**	(22;22)(q11;q11)	Mouth-Oropharynx: Squamous cell carcinoma	[96, 98]
**JAK2-BCR**	t(9;22)(p24;q11)	AML	[88, 96]
**PDGFRA-BCR**	t(4;22)(q12;q11)	CML	[88, 96]
**PRRC2B-BCR**	t(9;22)(q34;q11)	ALL	[101]
**RBM6-BCR**	t(3;22)(p21;q11)	Not reported	[88, 96]
**STYX1-BCR**	t(7;22)(q11;q11)	CML	[96, 102]

## BCR: THE PHILANDERING PARTNER

**Figure 1:**
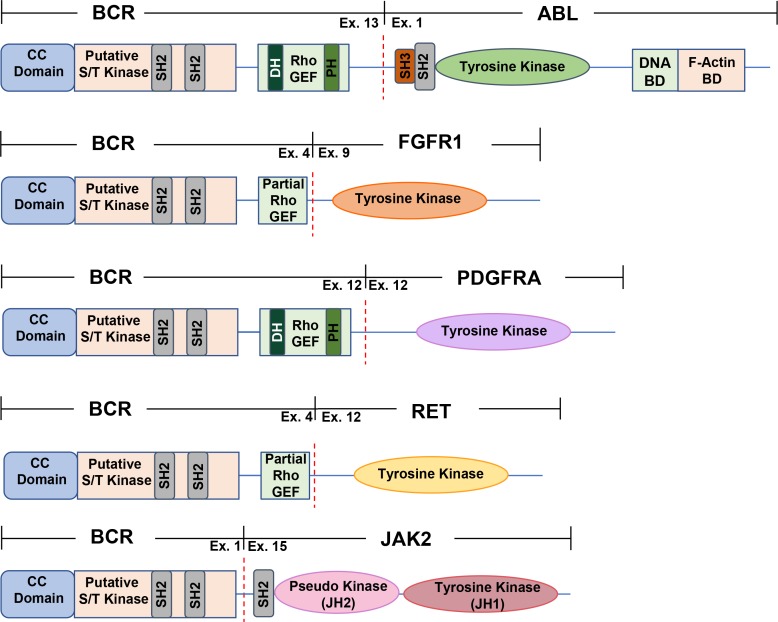
A schematic representation of commonly found BCR fusion proteins All fusions contain the coiled-coil domain found in BCR exon 1, fused to an activated kinase domain. All BCR fusions found in hematopoietic malignancies contain BCR as an N-terminal fusion partner. The dashed red line depicts the breakpoint for each fusion. The commonly found BCR-ABL p210 variant, BCR-FGFR1, BCR-PDGFRA, BCR-JAK2, and BCR-RET are all depicted above. CC domain, coiled-coil domain; putative S/T kinase, putative serine/threonine kinase domain; DNA BD, DNA binding domain; F-Actin BD, F-Actin binding domain; SH2, Src Homology 2 domain; SH3, Src Homology 3 domain; DH, Dbl homology domain; PH, Pleckstrin Homology domain; RhoGEF, guanine nucleotide exchange factor for Rho/Rac/Cdc42-like GTPases.

Since the discovery of the oncogenic fusion protein BCR-ABL, additional translocations with BCR as a fusion partner have been uncovered. Here, we discuss the fusions of BCR-ABL, BCR-FGFR1, BCR-PDGFRA, BCR-RET and BCR-JAK2 and their involvement in hematopoietic malignancies (Figure 1, Table 1). In addition to these well characterized fusion proteins, other BCR fusions have been discovered in solid tumors and hematological cancers, however these fusions have yet to be studied (Table 2). Although the reason behind the commonality of BCR as a fusion partner is not understood, we aim to discuss the mechanisms and current treatment options for cancers driven by these fusions.

### BCR-ABL fusion: The Philadelphia chromosome

The discovery of BCR-ABL was one the most influential findings for the treatment of hematopoietic malignancies, as this eventually identified the first target for specific TKIs, paving the way for directed drug therapies in patients. Nowell and Hungerford first discovered the Philadelphia chromosome, which encodes the BCR-ABL fusion protein in 1960, during the analysis of CML cases. The identification of the Philadelphia chromosome was a turning point, as this was the first demonstration of a chromosomal rearrangement being linked to a specific cancer [11]. Despite the discovery of BCR-ABL in 1960, it was not until 36 years later in 1996, when Imatinib was discovered to be an inhibitor of ABL and used to treat BCR-ABL positive CML [12, 13]. Since the initial characterization of BCR-ABL, the emergence of cancer genome sequencing has played a vital role in the detection of other translocation-induced malignancies. In fact, over 500 oncogenic translocations have been identified in hematopoietic cancers to date, again emphasizing the importance of identification and characterization of these oncogenic drivers for the development of finely tuned therapies for patients [14].

The Philadelphia chromosome results from the t(9;22)(q34;q11) translocation, which is detected in 95% of CML cases. CML is considered a three-stage disease described by an initial chronic phase where patients exhibit an expansion of the granulocytic cell lineage, typically lasting 3–4 years. Additional mutations can force the progression of CML into accelerated phase, followed by blast phase, which is characterized by the presence of 30% or more blast cells in peripheral blood or bone marrow [15]. Produced as a result of the Philadelphia chromosome, variants of the BCR-ABL gene fusion exist with alternative fusion points in either gene, which can be found in various leukemias [16]. The most commonly occurring BCR-ABL fusion is the p210 variant, where BCR exon 13 or 14 is found fused upstream of exon 1 to ABL; this variant is often found in CML (Figure 1). A BCR-ABL p190 variant, where BCR exon 1 is fused to ABL exon 2, is more frequently found in pediatric ALL and AML, and BCR-ABL p230, where BCR exon 19 is found exon 2 of ABL is seen in neutrophilic CML [16]. The p190 BCR-ABL variant characterizes a more acute leukemia usually of lymphoid origin, whereas the p210 BCR-ABL variant is a chronic leukemia of myeloid origin. Furthermore, p210 BCR-ABL is expressed primarily in early stages of myeloid maturation, with a decrease in expression seen with myeloid differentiation, suggesting that this disease is of stem cell origin [16]. It was recently uncovered that p210 and p190 BCR-ABL variants employ a differential signaling network to function within the cell. While the p210 variant saw a stronger activation of STAT5 and MAPK, the p190 variant activated Lyn kinase, as seen through quantitative comparative proteomic analysis [17]. The varying activation of kinase pathways by p210 and p190 suggests a different role of each variant as a driver of either myeloid or B-lymphoid transformation (Table 1) [17].

Interestingly, all variants contain BCR as a N-terminal fusion partner, fused to C-terminal ABL. All gene fusions also retain an intact BCR coiled-coil dimerization domain as well as a functional ABL kinase domain. It has been postulated that the coiled-coil domain from BCR facilitates the dimerization of ABL, thus activating its function. Furthermore, the coiled-coil domain in BCR also promotes the association of BCR-ABL with actin fibers, as BCR-ABL fusions lacking a coiled-coil domain had only a small increase in actin association. While ABL contains a C-terminal actin-binding domain in this gene fusion, mutations in either the coiled-coil domain of BCR or the C-terminal actin-binding domain in ABL attenuate the transformation ability of this fusion protein [15, 18].

**Figure 2:**
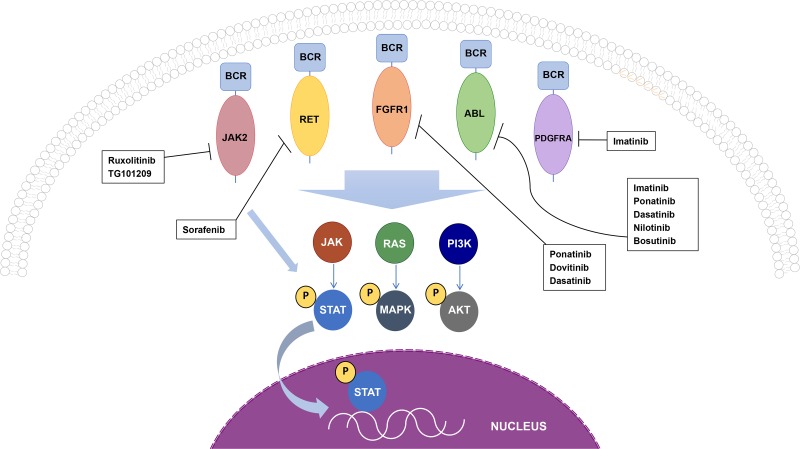
Oncogenic BCR fusion proteins and cellular signaling cascades BCR-ABL, BCR-FGFR1, and BCR-RET all activate STAT, MAPK, and AKT, while BCR-JAK2 only activates the STAT pathway, and signaling by BCR-PDGFRA remains to be elucidated. All drugs shown inhibit the kinase activity of each fusion protein and has been used in patients who are positive for the respective oncogenic fusion.

The BCR-ABL fusion exhibits cytoplasmic localization, and activation of the JAK/STAT, PI3K/AKT, and the RAS pathways (Figure 2). Specifically, the activation of STAT5 may contribute to the anti-apoptotic activity shown by patient derived BCR-ABL cell lines [16]. Additionally, BCR-ABL expression leads to IL-3 independent growth of Ba/F3 cells, despite the lack of secreted IL-3 detected in these cells [19]. Although ABL is a non-receptor kinase and usually displays low levels of constitutive kinase activity, the BCR-ABL fusion protein shows constitutively activated tyrosine kinase activity, attributed to the kinase domain in ABL. Furthermore, the extent of transforming activity is correlated to the degree of tyrosine kinase activity of BCR-ABL [16]. In addition, BCR-ABL is known to induce the tyrosine phosphorylation of Crkl, Shc, Syp, Fes, Vav, and paxillin proteins, suggesting a possible cell signaling or cell growth associated role for these interactions [16]. Endogenous BCR interacts with BCR-ABL and can form heterotetramers through the BCR coiled-coil domain. Furthermore, BCR binds to SH2 domains present in ABL, which is postulated to be functional feedback regulation, as serine phosphorylation within the kinase domain of BCR inhibits the kinase activity of ABL [16].

Altered mRNA translation and interaction between various upregulated genes have been shown to aid the cellular survival of BCR-ABL. In patient derived BCR-ABL positive cell lines, this fusion protein increased expression and activity of transcriptional inducer and translational regulator heterogeneous nuclear ribonucleoprotein K (HNRPK) through MAPK activation. Furthermore, the HNRPK/MAPK pathways have been demonstrated to control BCR-ABL activity through the regulation of myc mRNA translation [20]. In addition, long non-coding RNAs (lncRNAs) have been discovered to be involved in the progression of BCR-ABL positive CML [21, 22]. In particular, BCR-ABL mediated cell transformation requires the silencing of tumor suppressor, lncRNA-BGL3, which was shown to be suppressed through c-myc dependent DNA methylation [22]. In addition to gene overactivation or mutation, the misregulation of non-coding RNAs has been implicated in various cancers [23]. Non-coding RNAs are transcripts coded by the genome, which are not translated into protein. However, non-coding RNAs are known to regulate chromatin dynamics, gene expression, growth and development. Furthermore, alteration in lncRNA expression or mutation has been shown to promote malignant neoplasms [23]. In particular, lncRNAs have been discovered to be involved in the progression of BCR-ABL positive CML.

BCR-ABL has been shown to exhibit anti-apoptotic activity; the oncogenicity of BCR-ABL is facilitated by the suppression of apoptosis through the expression of the anti-apoptotic protein Bcl-2 [24]. Bcl-2 is a downstream target of the Ras pathway, and it is suggested that BCR-ABL regulation of Bcl-2 requires an active Ras signaling pathway [25]. A newly discovered interaction between scaffold protein AHI-1, BCR-ABL and Dynamin-2 (DNM-2) has been demonstrated to regulate the leukemic properties of hematopoietic stem cells (HSCs). This AHI-1/BCR-ABL/DNM-2 complex regulates HSCs through cellular endocytosis and ROS mediated autophagy, suggesting that this complex is a possible therapeutic target for the eradication of leukemic HSCs [26].

The extensive characterization of BCR-ABL has led to therapeutic advances for patients with Philadelphia chromosome positive CML. Although interferon alfa, hydroxyurea, or busulfan had typically been used to treat CML, these failed to achieve complete cytogenic response. However, the discovery and use of the potent TKI, imatinib, has led to significant advances in overall CML remission and has elicited hematologic and cytogenic response in a majority of patients [27]. The discovery of imatinib followed by the emergence of 2nd and 3rd generation TKIs, such as has dasatinib, nilotinib, and bosutinib has led to an increase in life expectancy of chronic-phase CML patients from 3–7 years to that similar to the general population [28]. However, the use of imatinib in patients has led to imatinib resistant CML malignancies in some cases. Mechanisms of imatinib resistance include point mutations in the ABL kinase domain, over expression of BCR-ABL, or up regulation of SRC kinase, which acts independently of BCR-ABL [29]. Although imatinib treatment remains the standard of care for Philadelphia chromosome positive CML, increasing imatinib resistance has led to 2nd generation TKIs including dasatinib, nilotinib, and bosutinib, which have shown efficacy in patients who developed BCR-ABL kinase domain mutations while receiving imatinib [29].

However, all 2^nd^ generation TKIs are inactive towards the BCR-ABL T351I mutation, a gatekeeper mutation commonly identified in imatinib resistant CML [29]. BCR-ABL T351I was the first imatinib resistant mutation detected in patients [30]. T351 in BCR-ABL controls the access of imatinib to a hydrophobic pocket in the kinase active site that does not contact ATP. However, substitution of T351 to a residue with bulkier side chains is a common mechanism of resistance for ATP-competitive kinase inhibitors [30]. Furthermore, the BCR-ABL T351I has been detected in imatinib naïve patients, and nearly 20% imatinib resistance is accounted for by this gatekeeper mutation. Thus, there exists a crucial need to develop additional therapies for the treatment of CML. Ponatinib, a 3rd generation TKI with activity against BCR-ABL T351I mutation, has shown promising results in patients [29]. Five year results from the ponatinib phase 2 PACE trial has shown that this 3rd generation TKI is effective in treating patients with relapsed or intolerant CML, Philadelphia chromosome positive ALL, or malignancies with BCR-ABL T351I [31]. Furthermore, these long-term results indicate that ponatinib demonstrates clinical value with long lasting responses in chronic phase CML patients, suggesting the use of ponatinib is beneficial in patients who are not sensitive to 1^st^ or 2^nd^ generation TKIs (Figure 2) [31].

Although TKIs are the first line of treatment for CML, many patients will require additional concurrent forms of treatment for complete remission [32]. The cellular function of BCR-ABL is dependent on the molecular chaperone Hsp90, suggesting that drugs which target this chaperone complex could be therapeutically beneficial [32]. Indeed, the inhibitor Aminoxyrone (AX), which targets Hsp90 dimerization via the Hsp90 C-terminal domain, has achieved success in inducing apoptosis in patient derived CML cell lines. These results indicate that C-terminal Hsp90 inhibition may be a therapeutic option for patients with other types of therapy-refractory leukemia. The analysis of the BCR-ABL fusion protein has led to new therapeutic advancements, which emphasize the importance of personalized medicine in healthcare, and the need for an increased understanding of these oncogenic fusions.

### BCR-FGFR1 fusion: The second most common fusion partner, and a receptor tyrosine kinase (RTK)

The fusion of BCR and fibroblast growth factor receptor 1 (FGFR1) arises from the t(8;22) (p11;q11) translocation, occurring commonly in EMS or stem cell leukemia (SCLL) but also observed in AML, atypical chronic myeloid leukemia (aCML) and B-cell lymphomas. This fusion, similar to other well characterized oncogenic BCR fusions, contains BCR as the N-terminal fusion partner (Figure 1). In addition, patients who are positive for BCR-FGFR1 often exhibit symptoms of leukocytosis [33]. Although patients who harbor FGFR1 rearrangements have a relatively poor prognosis, chemotherapy during blast crisis often allows regression to chronic phase after therapy [33]. Interestingly, most patients who had the t(8;22) (p11;q11) translocation had B lineage of the blast phase, indicating that the site of BCR breakpoint may play an important role in triggering B lineage [33].

While FGFR1 normally contains an extracellular immunoglobulin-like domain, a transmembrane domain, and a cytosolic kinase domain, this fusion gives rise to a putative kinase-kinase fusion product, with the putative serine-threonine kinase domain of BCR fused to the tyrosine kinase domain of FGFR1. Exon 4 of BCR has been found fused to exon 9 of FGFR1, with the RhoGEF domain in BCR partially intact in this fusion [34]. This fusion displays predominantly cytoplasmic localization, and the kinase domain of FGFR1 becomes constitutively activated, leading to the activation of STAT3, STAT5, AKT, MAPK, as well as IL-3 independent growth of Ba/F3 cells (Figure 2) [5]. Interestingly, similar to all other oncogenic BCR fusions, BCR-FGFR1 also retains the coiled-coil dimerization domain of BCR. This dimerization domain appears to be essential for the oncogenic activity of this fusion protein.

The RhoGEF domain in BCR is suggested to play an inhibitory role for BCR-FGFR1 oncogenicity. Loss of the GEF domain in this fusion increased leukemogenesis, enhanced cell proliferation, and promoted stem cell expansion and lymph node metastasis in mice [35]. In addition, deletion of the GEF domain suppressed the activation of RHOA and PTEN, leading to increased activation of AKT. Although the fundamental biochemical and oncogenic consequence of the BCR-FGFR1 fusion protein is clearly constitutive FGFR1 activation, deletion of the GEF domain in BCR is suggested to contribute as well through its suppression of RHOA signaling [35].

Furthermore, expression of various genes and miRNAs have been implicated in BCR-FGFR1 driven cancers. Cell lines derived from mouse models for leukemogenesis driven by BCR-FGFR1 have shown that high MYC expression is associated with constitutive expression of this fusion protein. Additionally, suppression of MYC function through interruption of the MYC-MAX complex halts cell cycle grown and enhanced apoptosis in Ba/F3 cells expressing BCR-FGFR1 [36]. While miRNAs have pathogenic roles in the progression of leukemias, the miR-17/92 cluster has been associated with the development of B lymphomas resulting from BCR-FGFR1 expression [37]. Forced expression of the miR-17/92 cluster resulted in cell proliferation, while inhibition resulted in reduced cell growth and apoptosis, indicating that the miR-17/92 cluster is a downstream effector of FGFR1 in BCR-FGFR1 driven leukemia [37]. Moreover, dynamic gene profile changes can accompany the progression of SCLL due to constitutive FGFR1 kinase activity, as studied in BCR-FGFR1 AML and SCLL mouse models [38]. SCLL is often characterized as a stem cell disease, where leukemic stem cells are usually considered an underlying cause to the resistance of chemotherapy. Numerous genes found in T-cell receptor function, T-cell development, migration, and activation were found inactivated in hematopoietic stem cells. In particular, transcription factors Zeb2, GFI1b, BCL11a, and IRF8A, which maintain normal hematopoietic stem cells, were found to be either inactivated, or suppressed in leukemic stem cells, suggesting that their down regulation may have important consequences for the development of BCR-FGFR1 driven AML [38].

Patients who harbor a BCR-FGFR1 gene arrangement have a relatively poor prognosis, with few treatment options available. Despite extensive chemotherapy, the only known curative option for patients is allogenic hematopoietic stem cell transplantation (HSCT) [39]. Patients who were treated with HSCT had a 77.8% complete remission rate with long-term disease free survival, even if residual disease was detected during the time of transplantation. However, patients who received HSCT from a matched sibling donor experienced disease relapse, suggesting a possible dependence on the transfusion-induced suppression of the host’s malignant cells, referred to as graft-vs-leukemia effect, for obtaining disease free survival for BCR-FGFR1 positive cancers. Due to the limited population of HSCT treated patients, the complete effect of transplantation remains to be uncovered [39]. Additionally, further characterization of BCR-FGFR1 has led to the use of several TKI therapies, which may be beneficial for patients either in search of a HSCT, or those not eligible for transplantation. Recently, TKIs dovitinib, ponatinib, and dasatinib were used to treat a patient who harbored the BCR-FGFR1 gene fusion (Figure 2). While dovitinib has a high specificity for FGFR1 inhibition, ponatinib has a more broad TKI effect, and dasatinib is readily clinically available. All three TKIs exhibited a growth inhibitory effect on primary EMS leukemic cells, indicating that these drugs may be therapeutically beneficial in patients who harbor a BCR-FGFR1 translocation [40]. The use of these novel RTK therapies against EMS yet again highlights the need for personalized medicine for the treatment of oncogenic gene fusion driven cancers.

### BCR-PDGFRA fusion: Another RTK fusion partner

Platelet-derived Growth Factor Receptor Alpha is encoded by one the of four genes in the PDGFR family, located on chromosome 4 [41]. When expressed in the immune system, it is often found in bone marrow, whole blood, white blood cells and lymph nodes, as well as thymus [10]. PDGFRs consist of 5 immunoglobin like/ligand binding domains, a juxtamembrane domain, a WW domain, as well as a kinase domain [41, 42]. Similar to other RTKs, upon ligand binding, PDGFR undergoes receptor dimerization, autophosphorylation, thereby activating downstream pathways including RAS, and JAK/STAT pathways [5]. Previous studies have shown that the WW domain, containing two conserved tryptophan residues, serves an autoinhibitory role in the juxtamembrane domain. Loss of the WW domain contributes to receptor constitutive activation, overactivation of downstream pathways, thereby leading to carcinogenesis [5, 42].

The fusion of BCR to PDGFRA is the second most common fusion protein involving PDGFRA. This BCR-PDGFRA fusion was first discovered in patients with aCML with a breakpoint of t(4;22) (q12;q11), fusing either exon 7or exon 12 of BCR to exon 12 of PDGFRA [43] (Figure 1). To date, this oncogenic fusion protein has been found in myeloproliferative neoplasms and T-cell lymphoblastic leukemia with alternative fusion points, joining BCR exon 7, 12 or 17 to PDGFRA exon 12 [44–46]. Within these fusions, BCR contains the intact oligomerization domain, putative serine/threonine kinase domain, and partial or complete GEF domain [44–46]. Resulting from this gene fusion is an oncogenic driver that preserves the N-terminal coiled-coil oligomerization domain of BCR followed by a truncated WW domain as well as an intact kinase domain provided of PDGFRA at the C-terminus [47]. It is possible that BCR-PDGFRA undergoes oligomerization using the N-terminal coiled-coil domain provided by BCR, thereby resulting in the constitutive activation of the PDGFRA kinase domain.

Currently, little is known about the localization of BCR-PDGFRA. However, prior studies on FIP1L1-PDGFRA, a similar gene fusion found in chronic eosinophilic leukemia conserving exon 12 of PDGFRA, suggested a cytoplasmic localization. As such, it was discovered that FIP1L1-PDGFRA overactivates the JAK/STAT5 pathway but not the Ras/MAPK pathway. Unlike wild type PDGFRA, the cytoplasmic localization of FIP1L1-PDGFRA prevents access to the farnesylated Ras, therefore unable to activate the MAPK pathway [47]. Due to the same conservation of exon 12 PDGFRA and the loss of the juxtamembrane, WW domain as well as the coiled-coil domain contributed by BCR, there exist a number of structural similarities between BCR-PDGFRA and FIP1L1-PDGFRA. Therefore, BCR-PDGFRA may share the same functional mechanisms and localization similar to FIP1L1-PDGFRA.

Previous studies have shown that by targeting the kinase domain of PDGFRA with the TKI imatinib, patients showed a decrease in BCR-PDGFRA expression and maintained a rapid, efficient response, indicating the efficacy of imatinib in targeting this oncogenic driver (Figure 2) [44]. This again emphasizes the need for targeted therapies in oncogenic BCR translocation-induced neoplasms [44].

### BCR-RET fusion: The RTK theme continues

The proto-oncogene RET (Rearranged during Transfection), a receptor tyrosine kinase, resides on human chromosome 10q11.2, and regulates cell survival, proliferation, and motility [48]. When expressed in the human immune system, the RET protein is often harbored in bone marrow, white blood cells, whole blood, and lymph nodes, as well as thymus [10]. RET contains an extracellular domain that contains four cadherin-like domains, followed by a transmembrane domain, and a tyrosine kinase domain [49]. Upon binding of the glial cell line-derived neurotrophic factor (GDNF) ligand family (GDNF, neurturin, artemin and persephin), RET undergoes receptor dimerization, autophosphorylation, followed by activation of downstream pathways including Ras/ERK, PI3K/AKT, as well as JAK/STAT [48, 50]. RET has vast implications in human diseases and is commonly discovered in the forms of gain-of-function and loss-of-function mutations and gene fusions, resulting directly in human pathogenesis such as Hirschsprung disease, papillary thyroid cancer (PTC) and chronic myelomonocytic leukemia (CMML) [49, 50].

The translocation of BCR to RET t(10;22)(q11;q11) was first discovered in patients with CMML [51]. It is a product of fusing exon 4 of BCR to exon 12 of RET, joining the coiled-coil oligomerization domain, serine/threonine kinase domain, and partial GEF domain of BCR with an intact kinase domain of RET (Figure 1) [16, 51] Following the initial discovery of this translocation, studies have revealed interleukin 3 (IL-3) independent growth using Ba/F3 cells and transforming activities using NIH3T3 cells upon transfection with BCR-RET, showing the carcinogenesis of this driver gene [51].

BCR-RET overactivates the Ras-ERK pathway, in addition to JAK/STAT3 and PI3K/AKT pathways [51]. Although imatinib has shown strong efficacy in targeting BCR-ABL in CML, patients exhibiting BCR-RET fusions have shown little response to imatinib. However, when treated with Sorafenib, a TKI targeting RET, patients have shown major hematological remission, demonstrating normal white blood counts (Figure 2) [51]. The use of these novel TKI therapies for specific translocations highlights the need for personalized medicine for the treatment of oncogenic gene fusion driven cancers.

### BCR-JAK2 fusion: A non-RTK fusion partner

The Janus kinase (JAK) family consists of four related non-receptor tyrosine kinases that transduce cytokine-mediated signals through the JAK-STAT pathway. Janus kinase 2 (JAK2), located on chromosome band 9p24, plays a crucial role in myelopoietic regulation [52, 53]. Upon binding of relevant cytokines, the cytokine receptor-JAK2 complex becomes activated, leading to progressive phosphorylation of the downstream STATs (Signal Transducer and Activator of Transcription), which translocate to the nucleus and regulate gene expression [53, 54]. To date, the known oncogenic associations of JAK2 in myeloproliferative neoplasms (MPNs) consist of either gain-of-function mutation or translocation [53, 55]. The most commonly found mutation in MPNs is V617F in JAK2, which disrupts the putative inhibitory role of the JH2 (JAK Homology) domain of JAK2 on the tyrosine kinase domain of JAK2 (JH1). As a result, the JAK2 kinase domain adopts an active conformation, therefore creating a constitutively activated JAK2 [56].

The oncogenic gene fusion BCR-JAK2 occurs rarely, with a few cases found in typical CML (chronic myeloid leukemia), AML (acute myeloid leukemia), ALL, (acute lymphoblastic leukemia), and B-cell lymphomas [52, 54, 55]. The most common BCR-JAK2 translocation is t(9;22)(p24;q11) [57]. These chimeric proteins show a fusion between BCR exon 1 to exon 19, 15, or 17 of JAK2. Despite the existence of various fusion points, all BCR-JAK2 fusions retain the intact N-terminal coiled-coil domain provided by BCR fused to the tyrosine protein kinase domain (JH1) from JAK2, suggesting a constitutively activated kinase domain caused by oligomerization of the coiled-coil domain of BCR (Figure 1) [52, 55].

Ba/F3 cell lines stably expressing BCR-JAK2 exhibit IL-3 independence and cytoplasmic localization of the BCR-JAK2 fusion protein. Furthermore, BCR-JAK2 expression led to enhanced activation of STAT5, as well as tumorigenesis when injected into mice [54]. *In vitro* experiments showed that treating the same Ba/F3 cells with TG101209, a JAK2 selective inhibitor, completely abolished the signaling activities of BCR-JAK2; additionally, flow cytometry data showed an increase in apoptosis [54]. Upon treatment with ruxolitinib, an FDA approved JAK1/JAK2 inhibitor, patients with BCR-JAK2 fusions initially showed complete remission followed by relapse in 12–18 months, indicating the limited efficacy of this option [57]. These results collectively suggest that therapeutic potential of JAK2 specific inhibitors to treat patients exhibiting BCR-JAK2 fusions (Figure 2).

## BCR: NORMAL STRUCTURE AND FUNCTION

BCR, also known as BCR1, RhoGEF and GTPase activating protein, is a protein-coding gene, which has been associated with 8p11 myeloproliferative syndrome (EMS), chronic myeloid leukemia (CML), and acute lymphoblastic leukemia (ALL). BCR was first identified fused to Abelson murine leukemia viral oncogene homolog-1 (ABL), also known as the Philadelphia chromosome. However, since then, BCR has been identified fused to Fibroblast Growth Factor Receptor 1 (FGFR1), Platelet Derived Growth Factor Receptor Alpha (PDGFRA), Ret Proto-Oncognene (RET), and Janus kinase 2 (JAK2). Interestingly, BCR fusion proteins that are drivers of cancer have only been identified in hematological cancers (Table 1). Although a common fusion partner, the endogenous function of the BCR gene remains unknown. Here, we seek to define the BCR gene in two ways; firstly, through its domains found in oncogenic fusion proteins, and secondly through unraveling the endogenous function of BCR.

### BCR domains commonly found in oncogenic fusion proteins

The BCR gene is located on chromosome 22q11, spans for 130kb and contains 23 total exons, with alternative exon 1 and exon 2, ultimately coding for a 1271 amino acid protein [16].

**Figure 3:**
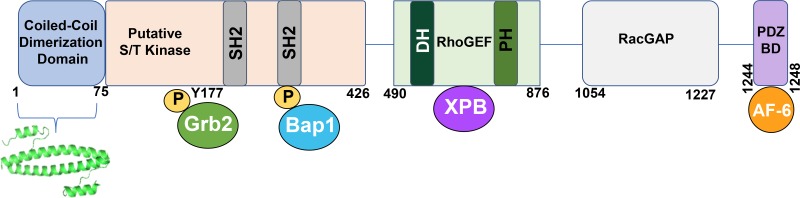
A schematic representation of the BCR protein BCR consists of an anti-parallel coiled-coil dimerization domain, within amino acids 1–75. Directly below is the crystal structure for this domain, depicted as a dimer (PDB 1K1F). The putative serine/ threonine kinase domain is portrayed through residue 426. This domain contains two SH2 binding sites, which interact with ABL SH2 domains. The adapter protein Grb2 binds to phosphorylated Y177 on BCR, and Bap1 also interacts with BCR via phosphorylated serine residues present in the second SH2 binding site. The RhoGEF domain is shown, containing Dbl Homology (DH) and Pleckstrin Homology (PH) domains, typical of a GEF. XPB associates with the GEF domain. The RacGAP domain encompasses amino acids 1054–1227, while the PDZ binding domain binds to AF-6 through the S-T-E-V motif found in the C-terminus of BCR. PDZ domains are named for three proteins that share the domain; Post synaptic density protein (PSD95), *Drosophila* Disc large tumor suppressor (Dlg1), and Zonula occludens-1 protein (zo-1). The associated proteins shown are: Grb2, Growth factor receptor-bound protein 2; Bap1, BRCA1 associated protein-1 (ubiquitin carboxy-terminal hydrolase); XPB, Xeroderma Pigmentosum type B (an ATP-dependent DNA helicase).

The structure of the BCR protein is varied with many domains (Figure 3). The first exon includes an oligomerization domain, putative serine/threonine kinase domain, a growth factor receptor bound protein 2 (Grb2) binding site, a BCR associated protein-1 (BAP-1) interacting site, and two SH2 domains. A central guanine nucleotide exchange factor (GEF) domain is encompassed by exons 3–8, followed by a RacGap domain found in exons 19–23 and PSD95, Dlg1, Zo-1 (PDZ) domain binding motif [16, 58]. (Figure 3).

BCR contains an anti-parallel coiled-coil oligomerization domain, which plays a crucial role in the kinase activity of its fusion partner [59, 60]. This dimerization domain is located on the N-terminus and spans from amino acids 1–75. Disruption of the coiled-coil domain either by insertion of a beta-turn sequence, or complete deletion of amino acids 1–75 results in a loss of transformation of BCR-ABL in rat-1 fibroblasts, indicating the importance of the BCR dimerization domain for transformation [60]. Additionally, inhibition of the dimerization domain in BCR has been accomplished by the use of a designed coiled coil, which preferentially binds to BCR-ABL than to itself. This peptidomimetic disrupts the dimerization of BCR, and thereby halts activation of the ABL kinase [61]. The contribution of a dimerization domain by BCR is critical in the transforming ability and activation of its fusion partner. Inhibition of the BCR oligomerization domain remains the only therapeutically targetable domain known in BCR to date. Furthermore, the contribution of a dimerization domain by BCR is critical in the transforming ability and activation of its fusion partner.

BCR contains a putative serine/threonine kinase, as well as two SH2 domains in exon 1. Although BCR has weak homology to other known serine/threonine kinases, BCR has been shown to autophosphorylate on serine and threonine residues and can phosphorylate both casein and histones [62]. Furthermore, cysteine 332 in BCR is essential for its kinase activity, as mutations that effect C332 abrogates its autophosphorylation activity [62].

BCR also contains two SH2 domains, which interact with ABL SH2 binding sites. These SH2 domains on BCR encompass amino acids 192–242 and 293–413 on BCR exon 1. Full length BCR binds specifically to the SH2 binding site on ABL, through phosphorylated serine and phosphorylated threonine residues [63]. Furthermore, BCR is known to interact with growth factor receptor bound protein2 (Grb2) at Y177 in BCR. This interaction is mediated by tyrosine phosphorylation where Grb2 SH2 domain interacts with Y177 on BCR [64]. Ablation of this interaction when BCR Y177 is mutated to phenylalanine significantly reduces Ras pathway activation as seen in BCR-ABL. BCR associated protein-1 (Bap-1), a 14-3-3 family member of the phospho-serine binding adapter proteins is shown to associate with BCR through sequences encoded in the first exon of BCR [65]. Tyrosine phosphorylation of BCR reduces association of Bap-1 with BCR [66].

The central GEF domain in BCR which spans amino acids 501–870 contains tandem Dbl Homology (DH) and Pleckstrin Homology (PH) domains, which are shared by all members of the RhoGEF family. The DH domain represents the catalytic core of the RhoGEF family, and BCR is known to catalyze the exchange of GDP for GTP on small GTPases Rac1, Cdc42, and RhoA [67]. Additionally, xeroderma pigmentosum group B (XPB), an ATP dependent helicase that is part of the TFIIH transcription factor complex has been shown to interact with the GEF domain in BCR. The binding of XPB to BCR in BCR-ABL reduced the ATPase and helicase activity of XPB, suggesting that the dysfunction of XPB may play a part in blastic crisis in CML [68]. Although BCR contains GEF activity in its central domain, it is a unique protein as it also contains GAP activity in its C-terminus, thus, possessing two opposing functions. Both BCR and ABR show GAP catalytic activity towards Rac and Cdc42, suggesting that BCR serves both as GEF and GAP for these GTPases [69].

Although the breakpoints for BCR fusion proteins in hematologic cancers vary, they all contain the coiled-coil dimerization domain present in BCR (Figure 3), indicating that the dimerization domain is vital for the oncogenic ability of these fusions. The coiled-coil domain in BCR is essential for cell transformation, as seen through assays performed with BCR-ABL [60]. In addition, BCR contributes this coiled-coil domain to BCR-FGFR1, BCR-PDGFRA, BCR-RET, and BCR-JAK2 (Figure 1). It is hypothesized that this oligomerization domain of BCR is necessary for the oncogenic activity of these other fusion proteins, however this remains to be elucidated.

### Unraveling the endogenous function of BCR

BCR is ubiquitously expressed, with the highest expression levels in brain and hematopoietic cells. It is expressed in the early stages of myeloid differentiation and these expression levels reduce significantly as cells mature. In addition to BCR, BCR related genes BCR2, BCR3, and BCR4, have also been mapped to chromosome 22q11. While these BCR related genes are not translated into protein, they all contain high sequence similarity to the last seven exons of protein coding BCR1 [70]. BCR2 is the closest in proximity to the centromere of chromosome 22, followed by BCR4, BCR1, and BCR3. BCR2 and BCR4 both have amplified loci in K652 cells, a leukemia cell line containing the BCR-ABL fusion, which indicates that they fall between the amplification unit of ABL locus on the Philadelphia chromosome. Active BCR related gene, ABR, is an additional BCR related gene, located on chromosome 17p13.3. ABR, unlike BCR2, BCR3, and BCR4, is transcriptionally active and contains both the GEF and GAP domains, located in the C-terminus of BCR, but lacks the putative serine/threonine kinase activity found in the N-terminus of BCR [71].

BCR is shown to act as a negative regulator of cell proliferation and oncogenic transformation [72]. BCR is shown to bind to AF-6 (Ras Association Family 6); this interaction is mediated via the PDZ domain of AF-6, which binds to the PDZ binding domain at the C-terminus of BCR encoded by the last four amino acids S-T-E-V. In addition, BCR, AF-6 and RAS have been shown to form a trimeric complex which is suggested to down regulate RAS mediated signaling at sites of cell-to-cell contact [72].

The complexity of the BCR protein is once again established through its function as both a GEF and GAP, as seen through its central domain and its C-terminus, respectfully. GEFs regulate the exchange of GDP for GTP, thereby activating GTPases, whereas GAPs hydrolyze GTP and turn off GTPase signaling [73].

Although BCR is most often characterized as part of the Philadelphia Chromosome, recently, both BCR and ABR have been identified as critical regulators of brain development. BCR and ABR mRNAs are highly expressed in the brain, and disruptions of BCR and ABR in mice leads to abnormalities in postnatal cerebellar development [74, 75]. Furthermore, BCR was shown to localize at excitatory synapses and mice deficient in BCR exhibited enhanced Rac1 activity and had impaired spatial and object recognition memory [76]. BCR is a known regulator of the Par-Tiam1 complex, which controls cell polarity. Loss of BCR in this complex resulted in faster, random migration, and polarity defects in astrocytes [77]. In addition, the adapter protein, Src homology 2 domain containing protein 5 (SH2D5) has been shown to bind to BCR and regulate levels of Rac1GTP. The phospho-tyrosine domain in SH2D5 binds to the NxxF motif in the N-terminal region of BCR [78]. This interaction is crucial for the regulation of Rac1-GTP levels, and is suggested to impact synaptic plasticity, which is necessary for learning and memory. These additional studies further confirm the multi-faceted role of BCR in the cell, in addition to its common occurrence as a fusion partner.

### The importance of stem cells in BCR-fusion hematopoietic cancers

CML is considered a stem cell disease, where leukemic stem cells maintain a population of chemotherapeutic resistant cells. Both BCR-ABL and BCR-FGFR1 driven hematopoietic malignancies are considered of stem cell origin, and it is speculated that this may be the same for BCR-PDGFRA, BCR-RET and BCR-JAK2 induced cancers as well, however this remains to be investigated.

In particular, BCR-ABL expression during development of embryonic stem cells causes expansion of multipotent and myeloid progenitors, which could be the potential cell of origin responding to BCR-ABL induced CML [79]. This progenitor expansion is due to a suppression of apoptotic pathways and an increase in anti-apoptotic protein BCL-XL [79]. Although imatinib therapy has improved the standard of care in CML patients, many patients harbor residual leukemic cells following the discontinuation of imatinib treatment [80]. Furthermore, it has been demonstrated that these leukemic stem and progenitor cells are not oncogene addicted, and do not respond to imatinib treatment, which proposes a difficult problem to overcome, highlighting the need for additional therapeutic strategies [80].

BCR-FGFR1 driven cancers are also considered to be of stem cell origin. Furthermore, genes found in T-cell receptor function, T-cell development, migration, and activation were found inactivated in hematopoietic stem cells, indicating that this suppression could drive BCR-FGFR1 induced AML [38].

The understanding of these BCR fusion protein induced stem cell cancers will give further insight for additional therapeutic advancements.

## AT THE FOREFRONT: T CELL THERAPY IN PH+ LEUKEMIAS

The discovery of novel TKIs against BCR-ABL along with HSCT and chemotherapy treatment has improved response rates and disease free survival in patients. However, many obstacles still remain in treating imatinib resistant patients or older patients who are often ineligible for HSCT, or TKI treatment [81]. Although treatment with ponatinib is promising in BCR-ABL T351I positive CML, ponatinib is often associated with arterial thrombotic events in older patients with known cardiovascular disease [82]. Furthermore, unlike CML patients, patients with BCR-ABL driven ALL often relapse, despite treatment with TKIs [83]. Recently, CD19 chimeric antigen receptor T-cell therapy (CAR-T) therapy, and Bi-specific T-cell engager (BiTE) therapies have shown promise in treating hematological malignancies that result from BCR fusion protein driven cancers [81–83].

CAR-T-cell therapy uses engineered T cells expressing chimeric antigen receptors to redirect antigen specificity in adoptive immunotherapy, and has been primarily used to treat leukemias and lymphomas [84]. CAR-T therapy has emerged as a potential therapeutic option for BCR-ABL driven malignancies as well [81]. Recently, three patients positive for BCR-ABL p190 ALL were able to receive a molecular or complete hematologic remission following treatment with T cell therapy [81]. Of these patients, two were also given imatinib or ponatinib in addition to T-cell therapy, indicating that both TKIs and CAR-T cell therapy may work together to achieve hematologic remission. Although CAR-T therapy has been investigated in BCR-ABL driven ALL to date, it is hypothesized that this line of T cell therapy will also be beneficial in additional BCR fusion-driven cancers.

Recently, BiTE therapies, a class of artificial bi-specific monoclonal antibodies, have shown promising results in treating BCR-ABL driven ALL [85]. BiTE therapies are antibodies that allow patients’ T cells to recognize malignant cells though the combination of a CD3 site and a CD19 site. Upon interacting with the BiTE at the CD3 site, a T cell is then activated and is allowed to exert a cytotoxic response on the CD19+ target [86]. Specifically, blinatumomab, has shown promising results in treating Philadelphia chromosome driven B-ALL [85]. Patients were treated with blinatumomab with concurrent TKI treatment, and 8 of 9 patients were able to achieve complete molecular response [85]. Furthermore, blinatumomab has shown efficacy in treating patients with Ph+ ALL, as seen through a phase II multicenter study [87]. When 45 patients were treated with blinatumomab, 16 achieved complete remission, including 4 patients with the T315I mutation, indicating that this treatment exhibited antileukemic activity in patients with relapsed or TKI resistant ALL [87].

Although much headway has been made in treating Ph+ CML, additional therapies are necessary for patients who are TKI refractory or unable to tolerate current therapies due to age, or comorbidities. Thus, the use of T cell therapies for treating BCR-ABL induced malignancies is a promising therapeutic advance in tackling these problems. Although these therapies have only been investigated in Ph+ cancers to date, it is speculated that both CAR-T therapy and BiTE therapy will be beneficial in treating additional BCR fusion driven cancers.

## CONCLUSIONS

The emergence of personalized medicine and cancer genome sequencing has led to the discovery of chromosomal translocations, which are capable of producing an oncogenic protein. Of these translocations, BCR has been identified as a common fusion partner in hematopoietic cancers with over 5 known fusion partners to date. Although the reason behind commonality of BCR as a fusion partner is not well understood, it is speculated that these BCR fusions result from proximity to chromosomal fragile sites. Notably, BCR contributes a coiled-coil dimerization domain to all fusions discussed in this review, suggesting the importance of this domain for the oncogenic potential of these fusions.

The initial discovery of BCR fusion proteins led to the impactful role of personalized medicine in patient care. BCR-ABL, in particular was identified as the first target for TKI therapy, which opened up the door for targeted therapies in translocation induced cancers. Although the use of these targeted therapies is beneficial in various cancers, many obstacles remain due to relapse or drug resistance in patients. Therefore, additional approaches will be required for the characterization and treatment of translocation induced cancers. The identification of oncogenic BCR fusion proteins emphasizes the importance of determining malignant genetic alterations in patients and stresses the need for the development of personalized medical treatments for hematopoietic cancers.

## Abbreviations

ABL: Abelson murine Leukemia viral oncogene homolog-1
aCML: atypical Chronic Myeloid Leukemia
AF-6: Ras Association Family 6
ALL: acute lymphoblastic leukemia
AML: Acute Myeloid Leukemia
BAP: BRCA1 associated protein-1 (ubiquitin carboxy-terminal hydrolase)
BCR: Breakpoint Cluster Region
CML: Chronic Myeloid Leukemia
CMML: Chronic Myelomonocytic Leukemia
EMS: 8p11 Myeloproliferative Syndrome
FGFR1: Fibroblast Growth Factor Receptor 1
Grb2: Growth factor receptor-bound protein 2
HSCT: allogeneic Hematopoietic Stem Cell Transplant
JAK2: Janus Kinase 2
PDGFRA: Platelet Derived Growth Factor Receptor Alpha
PDZ: named for Post synaptic density protein (PSD95), *Drosophila* Disc large tumor suppressor (Dlg1), and Zonula occludens-1 protein (zo-1)
RET: RET proto-oncogene, “REarranged during Transfection”
RacGAP: Rac GTPase-activating protein
RhoGEF: guanine nucleotide exchange factor for Rho/Rac/Cdc42-like GTPases
SH2: Src Homology 2 domain
SH3: Src Homology 3 domain
TKI: Tyrosine Kinase Inhibitor
XPB: Xeroderma Pigmentosum type B (an ATP-dependent DNA helicase)
